# A High Performing Biomarker Signature for Detecting Early-Stage Pancreatic Ductal Adenocarcinoma in High-Risk Individuals

**DOI:** 10.3390/cancers17111866

**Published:** 2025-06-02

**Authors:** Norma A. Palma, Aimee L. Lucas, Bryson W. Katona, Alcibiade Athanasiou, Natasha M. Kureshi, Lisa Ford, Thomas Keller, Stephen Weber, Ralph Schiess, Thomas King, Diane M. Simeone, Randall Brand

**Affiliations:** 1 Immunovia, Inc., Durham, NC 27713, USA; 2 Division of Gastroenterology and Hepatology, Mount Sinai West and Sinai Morningside Hospitals, Icahn School of Medicine at Mount Sinai, New York, NY 10029, USA; 3 Department of Gastroenterology, University of Pennsylvania Perelman School of Medicine, Philadelphia, PA 19104, USA; 4 Proteomedix AG, 8952 Schlieren, Switzerland; 5 ACOMED Statistic, 04109 Leipzig, Germany; 6 Department of Surgery, University of California, San Diego Health, Moores Cancer Center, San Diego, CA 92037, USA; 7 Division of Gastroenterology, Hepatology and Nutrition, University of Pittsburgh Medical Center, Pittsburgh, PA 15213, USA

**Keywords:** pancreatic ductal adenocarcinoma, biomarkers, high-risk, surveillance, potentially resectable, familial pancreatic cancer, germline mutations

## Abstract

**Background/Objectives:** Early detection of pancreatic cancer can improve patient survival, and blood-based biomarkers to aid in this are a significant need. The goal of this study was to develop and evaluate the performance of a 4- to 6-plex biomarker signature for detection of early-stage pancreatic ductal adenocarcinoma (PDAC) that performs well in high-risk controls. **Methods**: Enzyme-linked immunosorbent assays were used to measure 10 previously identified serum protein biomarker candidates in Stage I and II PDAC cases (*n* = 128), high-risk controls (*n* = 465), and normal-risk controls (*n* = 30). Various combinations of biomarker candidates (models) were trained using machine learning and tested for robustness in differentiating cases from controls on the full cohort and in clinically relevant sub-types including those with diabetes, those ≥65 years of age, and low producers of carbohydrate antigen 19-9 (CA 19-9). **Results**: At 98% specificity, the top performing model, which was comprised of tissue inhibitor of metalloproteinase 1 (TIMP1), intracellular adhesion molecule 1 (ICAM1), thrombospondin 1 (THBS1), cathepsin D (CTSD), and CA 19-9, achieved 85% sensitivity in the full cohort and sensitivities of 91% in diabetics, 90% in ≥65 years of age, and 60% in low CA 19-9 producers. This model demonstrated significantly higher sensitivity in detecting PDAC in the full cohort and all sub-populations compared to CA 19-9 alone (*p* < 0.001). **Conclusions**: Our findings demonstrate the feasibility of a blood-based assay for detecting early-stage PDAC in high-risk individuals and key sub-populations, representing an important step towards improving diagnostic success for early-stage disease.

## Simple Summary

Using machine learning, this study identified a 5-plex serum-based biomarker signature with high sensitivity and specificity for detecting pancreatic ductal adenocarcinoma (PDAC) in a cohort of Stage I and II cases and high-risk controls undergoing surveillance. To date, no blood-based biomarkers have been approved specifically for PDAC surveillance or to aid in its diagnosis. Underrepresentation of high-risk controls in study populations has been cited as a primary reason for the failure of PDAC biomarkers in clinical trials. By addressing this shortcoming in our study cohort, our findings demonstrate a blood-based test for detection of early-stage PDAC that performs well in the clinical population likely to gain the most benefit from its use. Overall, these findings represent an important step towards improving early diagnostic success and extending survival of this devastating disease.

## 1. Introduction

Pancreatic ductal adenocarcinoma (PDAC) is a highly aggressive malignancy that claims approximately 50,000 lives each year and currently ranks as the third leading cause of cancer-related death in the United States [1]. Despite advancements in the understanding of PDAC biology and efforts to develop new therapies, its incidence continues to rise, and the average 5-year survival rate across all stages combined is only 13% [2,3].

PDAC usually remains asymptomatic until it has advanced to later stages, when treatments are considerably less effective [4,5]. Early detection of disease impacts patient outcomes such that diagnosis at Stage I or II results in a 44% 5-year survival rate versus a 3.1% 5-year survival rate, where surgical intervention is no longer an option [6]. The low incidence of PDAC in the general population limits recommended surveillance to those who carry a germline mutation that puts them at high risk for PDAC (i.e., *CDKN2A*, *STK11*, *BRCA2*, etc.), have a family history meeting the definition of familial pancreatic cancer, or have a mucinous pancreatic cyst [7,8,9]. Unfortunately, the most widely used surveillance tests, which include computed tomography (CT), magnetic resonance imaging (MRI), and endoscopic ultrasound (EUS), have limited sensitivity against small, early-stage tumors [10]. Carbohydrate antigen 19-9 (CA 19-9), the one biomarker approved for use in PDAC patients [11], can inform response to treatment but lacks the necessary sensitivity or specificity to be recommended for diagnosis or surveillance [12,13]. Additionally, 5–10% of the general population and up to 25% of some ethnic populations harbor homozygous alterations in the *FUT2/3* genes, which renders them incapable of producing and/or secreting CA 19-9 [14,15]. These individuals, as well as those with *FUT2/3* variants that result in low, but not nil, production of CA 19-9 are disadvantaged by relying solely on the use of this biomarker. Other populations, including diabetics and the elderly are also disadvantaged by relying on CA 19-9 because its levels tend to increase in response to both aging and diabetes [16,17].

To improve upon existing diagnostic tools and extend survival rates, significant efforts have been made to identify noninvasive biomarkers for detecting early-stage PDAC [18,19,20,21]. Although many biomarker candidates have been published, none have been approved specifically to aid in the diagnosis of PDAC, emphasizing the importance of continuing these efforts.

In a previous study, we evaluated candidate biomarkers for early-stage PDAC using Olink multiplex technology and targeted immunoassays in a cohort of Stage I and II PDAC cases and age- and sex-matched controls [22]. This study identified 41 serum-based proteins that differentiated PDAC from controls (*p* < 0.05). The primary endpoint of the current study was to develop a four- to six-plex biomarker signature from this initial pool of 41 candidates then assess its ability to discern early-stage PDAC in a genetic/familial high-risk patient population. Secondary study endpoints included developing a model that outperformed CA 19-9 alone in the full cohort and sub-populations studied.

## 2. Materials and Methods

### 2.1. Study Design and Participants

This retrospective study was conducted according to protocols that adhered to ethical standards outlined in the Declaration of Helsinki and were approved by the Institutional Review Boards/Ethics Committees of all 12 participating study sites before the study began (Supplemental Table S1). All participants provided written informed consent. The study complied with all relevant ethical regulations for research involving human participants. Banked serum samples were blinded for their clinical information using a unique clinical identifier such that no patient data or disease status was known by laboratory personnel until after protein biomarkers had been measured and the data set had been locked. The study population included: (1) patients recently diagnosed with Stage I or II PDAC (*n* = 128) (2) high-risk controls identified through PDAC surveillance programs (*n* = 465), and (3) normal-risk controls (*n* = 30). In this manuscript high-risk and normal-risk controls are collectively referred to as controls. Samples were retrospectively selected according to PDAC status from different centers and therefore were not randomized in relation to the screening population. The presence of diabetes (regardless of duration of diagnosis), age, and CA 19-9 status were evaluated in the groups examined. Where indicated, diabetics, those ≥65 years of age, and low CA 19-9 producing groups (<37 U/mL) were evaluated as sub-populations. 65 years was chosen as the cutoff to define older study participants because it would allow data from the Medicare population to be evaluated. Inclusion and exclusion criteria for study participation and methods used to select samples for the study are shown in Supplemental Figure S1.

### 2.2. Candidate Protein Selection and Measurement

From the 41 biomarker candidates identified in our previous study [22], 10 were selected for follow-up analysis based on their reported roles in PDAC, ability to distinguish PDAC cases from controls in the previous study, and/or the availability of protein-specific immunoassay materials that meet technical suitability for clinical use. CA 19-9 was also included for analysis due to its status as the only FDA-approved marker for PDAC patients. We targeted a 4- to 6-plex biomarker panel in ELISA format to ensure a reproducible and efficient application in clinical testing. CA 19-9 was measured by Dr. Risch Services AG (Buchs, Switzerland) using a COBAS 8000 modular analyzer (Roche Diagnostics, Rotkreuz, Switzerland) according to manufacturer’s instructions. ELISAs were optimized and the following proteins were quantitated by Proteomedix (Schlieren-Zurich, Switzerland): carboxypeptidase B1 (CPB1) (Aviva Systems Biology, San Diego, CA, USA #OKEH07759, RRID:AB_3678911), cathepsin D (CTSD) (Proteomedix AG, Schlieren, Switzerland #Proclarix^®^ CTSD ASSAY), intracellular adhesion molecule-1 (ICAM1) (Proteomedix, AG, # ICAM1), tissue inhibitor of metalloproteinases 1 (TIMP1) (Biotechne R&D Systems, Minneapolis, MN, USA #MAB97, RRID:AB_3678914 (capture antibody), #BAF970, RRID:AB_356824 (detection antibody), #970-TM (protein)), thrombospondin 1 (THBS1) (Proteomedix AG, Schlieren, Switzerland #Proclarix^®^ THBS1 ASSAY), glycoprotein non-metastatic melanoma protein B (GPNMB) (Biotechne R&D Systems, Minneapolis, MN, USA #DY2550, RRID:AB3678917), kallikrein related peptidase 10 (KLK10) (Aviva, San Diego, CA, USA #OKDD04348, RRID:AB_3678918), mer tyrosine kinase (MERTK) (LS-Bio, #LS-F462, RRID:3678919), phospholipase A2 group 1B (PLA2G1B) (LS-Bio, Seattle, WA, USA #LS-F33272, RRID:AB_3678920), and latent transforming growth factor-β binding protein 2 (LTBP2) (Abcam, Waltham, MA, USA #ab13988, RRID:AB_300815).

### 2.3. Statistical Analyses

A sample size estimation was performed at 80% power and an assumed disease prevalence of 20% as described [23]. The relationship between individual protein biomarkers and PDAC diagnostic status was evaluated using Mann–Whitney U tests. The training of the algorithm was performed as previously described [22]. Briefly, markers were used to train generalized linear models with embedded feature selection that included all possible 2- to 6-plex combinations and relevant imputations of candidate proteins using PDAC diagnosis as the binary outcome. All models were trained and cross validated on the full cohort and tested for overfitting using a 7 to 10 ratio of training/test splits from the full cohort. Their performance was then evaluated on the diabetic, ≥65 years, and low CA 19-9 producer sub-populations based on the sensitivity they achieved at 98% specificity. Area under receiver operating characteristic (ROC) curves (AUC) for the final model and its two variations were compared using the Delong method [24]. Sensitivities and specificities were compared using the McNemar test [25] or two-tailed student’s *t*-tests. For all tests, *p*-values < 0.05 were considered statistically significant. All statistical analyses were performed in R, version 4.4.1., RRID:PRJ_0000000000 [26].

## 3. Results

### 3.1. Patient Characteristics

Serum samples from 623 study participants (*n* = 128 cases, *n* = 495 controls) were analyzed. Demographics of the study population are shown in Table 1 (full cohort) and Supplemental Table S2 (sub-populations). The cohort’s median age was 64 years (SD: ± 8 years), with cases (68 years, SD: ± 10 years) being older than controls (63 years, SD: ± 7 years). No significant differences in the expression of final candidate proteins based on age were found (Supplemental Figure S2).

A disparity in sex distribution was observed: 379 participants (61%) were female and 244 (39%) were male. This was largely due to the female predominance of control subjects (65% female vs. 35% male) with a smaller male predominance in cases (46% female vs. 54% male). Sex disparities result from the typical composition of high-risk surveillance programs (predominantly female) [27] and the somewhat higher incidence of PDAC in males [28,29]. One of the candidate proteins, CBP1, demonstrated a significant difference in serum concentration between male and female controls and was eliminated from the final model selection. The final biomarkers used in the models showed no difference in expression between male and female controls or between male and female cases (Supplemental Figure S3).

### 3.2. Protein Analysis and Selection of Final Candidates

Using commercially available materials and reagents from various manufacturers, we developed ELISAs to quantitate 10 pre-selected biomarker candidates identified in our discovery study [22]: CTSD, CPB1, ICAM1, TIMP1, GPNMB, MERTK, PLA2G1B, KLK10, LTBP2, and THBS1. Due to poor analyte stability, high lot-to-lot variability of the assay, and/or extensive covariance with other potential final model candidates KLK10, MERTK, GPNMB, and PLA2G1B were excluded from further analysis.

We characterized expression of the remaining 6 proteins and CA 19-9 by comparing their serum concentrations between PDAC cases and controls. The abundance of every analyte except THBS1 was significantly different between the two groups (*p* < 0.001) (Table 2). The expression pattern of each analyte matched those observed in our previous biomarker discovery study (Supplemental Table S3). We next evaluated the diagnostic performance of the individual markers. At 98% specificity, CTSD, TIMP1, ICAM1, and CA 19-9 achieved 31–65% sensitivity while LTBP2, CPB1, and THBS1 achieved 2–12% sensitivity (Table 2). Compared to their performance in the full cohort, each analyte performed similarly or better in the diabetic, ≥65 years, and low CA 19-9 sub-populations (Supplemental Table S4).

**Figure 1:**
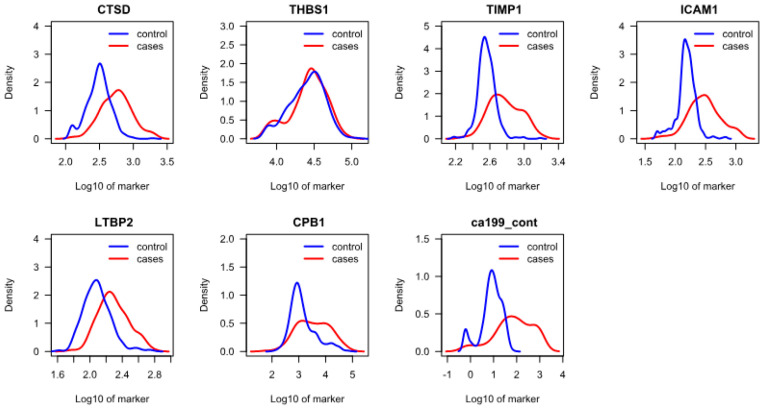
Density plots showing distribution of analyte concentrations in all cases and controls (full cohort).

Next, we compared the distribution of protein levels between all cases and all controls. CTSD, TIMP1, ICAM1, CPB1, LTBP2, and CA 19-9 demonstrated clear separation, while THBS1 demonstrated nearly complete overlap between the two groups, showing that its amounts were not significantly different between cases and controls when analyzed in the full cohort (Figure 1). However, in the diabetic sub-population THBS1 demonstrated a significant separation in its distribution (*p* = 0.031) (Supplemental Table S4 and Supplemental Figure S4), suggesting that it could be helpful in detecting PDAC in this sub-group despite its low overall sensitivity. For this reason, THBS1 was included in the model development studies.

### 3.3. Model Development and Cross Validation

To identify which biomarker signatures (i.e., models) achieve the best diagnostic performance, we used machine learning to train all possible 2- to 6-plex combinations of ICAM1, THBS1, TIMP1, CTSD, LTBP2, and CPB1 with and without CA 19-9 on the full cohort.

**Figure 2:**
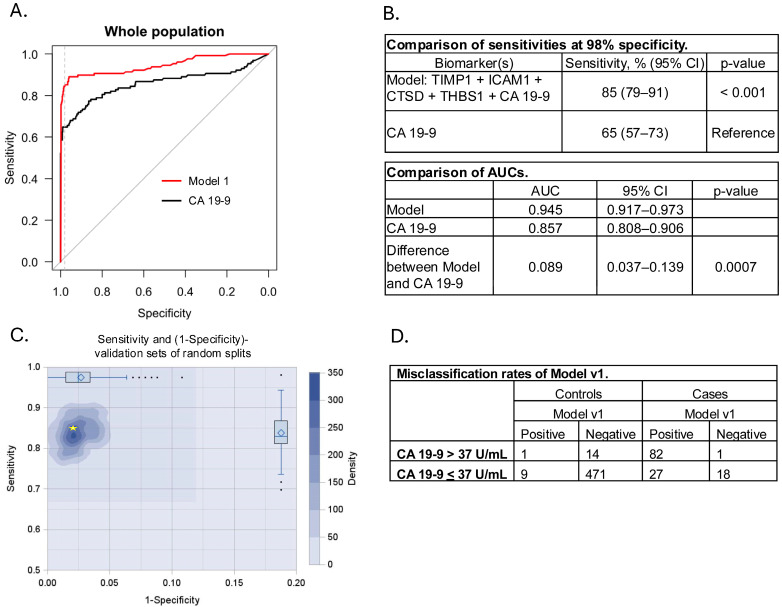
Model v1 performance and robustness in the full cohort. (**A**) ROC curves of the top performing model and CA 19-9 only. Gray line (comparator) depicts no model discrimination between groups (AUC = 0.5). (**B**) **Top**, Comparison of model sensitivity at 98% specificity presented as a percentage with a 95% CI. *p*-value according to McNemar indicates the difference in model sensitivity compared to CA 19-9 alone. **Bottom**, Comparison of AUCs for the Model and CA 19-9 showing 95% CIs, the difference between the Model and CA 19-9 AUCs, 95% CI, and associated *p*-value. (**C**) Density plot and boxplots of the 1000x random splits showing robustness of Model v1. The yellow star indicates model performance when trained using the full cohort. (**D**) Table showing the number of cases and controls that were misclassified by Model v1 and their CA 19-9 status.

The top performing model (Model v1), which was comprised of TIMP1, ICAM1, CTSD, THBS1, and CA 19-9, achieved 85% sensitivity at 98% specificity (95% CI) (Figure 2A,B) compared to 65% (95% CI) achieved by CA 19-9 alone (*p* < 0.001) (Figure 2B). We assessed the robustness of the model using a standard repeated data split and cross-validation strategy. Random training and validation splits (7 to 10 ratio) were repeated 1,000 times for each model. Boxplot sensitivities for the model were evaluated on the full cohort. Sensitivity of the training cohort at 98% specificity consistently fell within the range of performance of the validation cohort from the random splits across the model (Figure 2C), suggesting that the model was not over-fitted. Overall, this model (Model v1) diagnosed 10 of the 495 controls as PDAC positive (2% false positive rate) and 19 of the 128 PDAC cases as negative (15% false negative rate) (Figure 2D). Two variations of this model, which lacked either THBS1 (Model v2) or CTSD (Model v3) demonstrated similar performance (Supplemental Figure S5).

### 3.4. Model Performance in Sub-Populations

**Figure 3:**
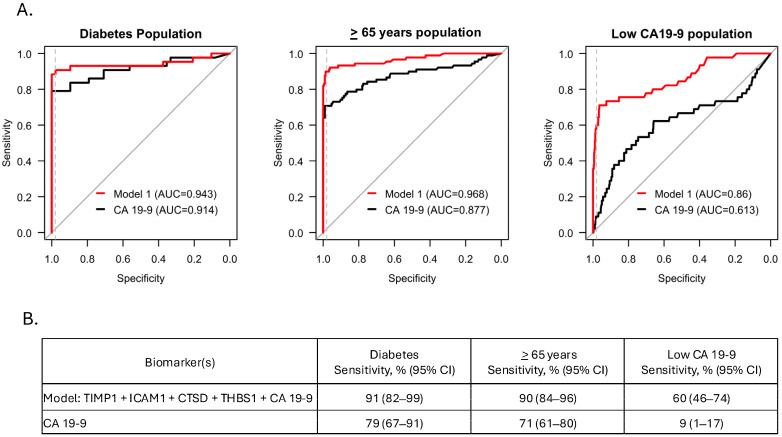
Model performance on sub-populations. (**A**) ROC curves showing model performance in the diabetic, ≥ 65 years, and low CA 19-9 sub-populations. Gray lines (comparators) depict no model discrimination between groups (AUC = 0.5). Dashed gray lines represent 90% specificity. (**B**) Sensitivities at 98% specificity are presented as a percentage with a 95% CI. Sensitivity achieved by Model v1 in all sub-populations compared to CA 19-9 alone, *p* < 0.001.

We next assessed the performance of Model v1 in the three pre-defined sub-populations. In the diabetic and ≥65 years groups, the model achieved sensitivities of 91% and 90%, respectively, at 98% specificity (95% CI), which outperformed CA 19-9 alone by 12 percentage points in the diabetic group and 19 percentage points in the ≥65 group (*p* < 0.001) (Figure 3). In the low CA 19-9 group, the model achieved 60% sensitivity at 98% specificity (95% CI), significantly outperforming CA 19-9 alone (9% sensitivity) at 98% specificity (*p* < 0.001) (Figure 3).

## 4. Discussion

A blood-based biomarker assay capable of detecting early-stage PDAC could address a significant clinical need. CA 19-9 alone lacks adequate sensitivity and specificity to serve as a surveillance marker and routine imaging to screen for PDAC is not recommended for the general population [30]. Furthermore, currently available diagnostic imaging techniques have limited sensitivity for detecting small, early-stage tumors. Our goal is to develop a noninvasive test that can aid in the detection of early-stage PDAC by implementing biomarkers that have robust sensitivity and specificity against Stage I and II disease in those at high risk for PDAC and in sub-populations that may be challenging to diagnose.

Here, we identified a model comprised of TIMP1, ICAM1, CTSD, THBS1, and CA 19-9 that distinguished PDAC cases from controls with a sensitivity of 85% at 98% specificity, thereby achieving the primary endpoint of this study. At 98% specificity this model also achieved sensitivities of 91%, 90%, and 60% in diabetics, those ≥65 years of age, and low CA 19-9 producers, respectively, to outperform CA 19-9 alone in both the full cohort and all clinical sub-populations, thereby achieving the secondary study endpoints. Two variations of the model that omitted either THBS1 or CTSD showed comparable performance, indicating that an abbreviated signature comprised of 4 biomarkers may be sufficient for clinical use.

**Figure 4:**
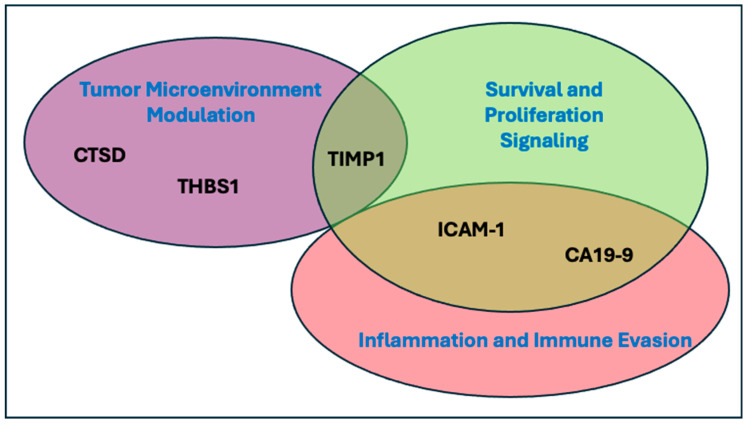
**Signature analytes and their role in pancreatic cancer**. CTSD = cathepsin D, THBS1 = thrombospondin 1, TIMP1 = tissue inhibitor of metalloproteinase 1, ICAM-1 = intracellular adhesion molecule 1, CA 19-9 = carbohydrate antigen 19-9.

TIMP1, ICAM1, CTSD, THBS1, and CA 19-9 are differentially expressed in PDAC tumors compared to noncancerous tissue and each play various roles in PDAC pathophysiology (Figure 4). High expression of TIMP1 and ICAM1 is associated with poor prognosis in PDAC patients [31,32,33,34], and both proteins are thought to promote metastasis by facilitating neutrophil extracellular trap (NET) formation as well as neutrophil and macrophage infiltration into the pancreas and liver where they create a premetastatic niche for tumors [35,36,37]. Like TIMP1 and ICAM1, CTSD is typically overexpressed in PDAC and has been implicated in cancer cell proliferation, migration, and tumor invasion [38,39]. THBS1 has been shown to both suppress and promote angiogenesis in PDAC through mechanisms that are currently under investigation [40,41,42]. THBS1 also promotes tumor metastasis in post-dormancy stages of PDAC by converting latent TGF-β1 complexes to their active form through loss of the tumor suppressor Smad4 and upregulation of MMP-9 production [43,44].

Due to their roles in PDAC progression TIMP1, ICAM1, CTSD, and THBS1 have all been previously evaluated as diagnostic biomarkers. Several reports show that incorporating any one of these proteins into diverse biomarker signatures, some of which include CA 19-9, improves diagnostic success over CA 19-9 alone [31,36,45,46,47]. The findings in the current study agree with these previous reports. Unlike previous studies our study focused on early-stage, potentially resectable PDAC cases and investigated diagnostic performance in both high-risk individuals undergoing surveillance and clinically relevant sub-populations. A key finding of our study is a set of biomarkers that performs well in not only the general PDAC population, but also in genetic/familial high-risk surveillance populations, and sub-populations that face diagnostic challenges, especially low CA 19-9 producers.

Investigators have attempted to improve diagnostic success in low CA 19-9 producers by identifying alternative diagnostic biomarkers [48,49] and establishing different clinical cutoff levels for these individuals relative to the general population [50]. Thus far, both approaches have had limited success. The performance of our model in the low CA 19-9 group is particularly noteworthy because for the first time it demonstrates a clinically useful, non-invasive test for early-stage PDAC patients who do not overexpress CA 19-9.

Although our findings are promising, we acknowledge this study had limitations. Due to the retrospective nature of the sample collection, the status of certain clinical variables, including biliary obstruction and pancreatitis at the time of sample collection are unknown, which prevented evaluation of the model’s specificity in these clinical contexts. This aspect will be evaluated in follow-up studies. Another limitation was the low racial/ethnic diversity in the test population, which remains a challenge in both clinical trials and high-risk pancreatic cancer surveillance [51]. We are addressing this issue in our current work by targeting study populations that more closely resemble the ethnic composition of the United States. As a third limitation, we note that, to appropriately power the study, 10 case samples that had been in storage significantly longer than the rest of the cohort (up to 10 years) had to be included in analysis. Of these, four were classified as false negatives (sensitivity 60%) suggesting that long-term sample storage may adversely affect test performance. This possibility is not surprising considering serum proteins are known to degrade over time even when stored at −80 °C. We are testing the effect of long-term storage in ongoing validation studies by comparing test sensitivity and specificity between the numerically oldest (50%) and newest (50%) cases and controls. We note that prolonged sample storage would not impact clinical utilization of this test because in practice, only recently collected samples would be analyzed. Finally, we emphasize that although statistical evaluation supports the robustness of this model, it is essential to validate its clinical performance with an independent set of cases and controls before its clinical implementation, and these validation studies are currently underway.

## 5. Conclusions

Our findings demonstrate a robust model with high sensitivity and specificity in differentiating Stage I and II PDAC cases from high-risk and normal-risk controls. The model also performs well in diabetic, ≥65 years, and low CA 19-9 producing sub-populations. Overall, these results justify advancement to the next phase of analytical and independent clinical validation studies. This biomarker panel represents an important step towards improving detection of early-stage PDAC and survival outcomes for this devastating disease.

## Figures and Tables

**Table 1:** Study participant demographics, full cohort.

Clinical Variable	All (n = 623)	Cases (n = 128)	Controls (n = 495)
Age, median (min-max)	64 (40–95)	68 (46–95)	63 (40–83)
CA 19-9 U/mL, median (min-max)	10 (0.6–1000) *	78 (0.6–1000)	9 (0.6–70)
Sex male, n (% of column heading)	244 (39%)	69 (54%)	175 (35%)
PDAC Stage I, n (% of column heading)	88 (14%)	88 (69%)	N/A
PDAC Stage II, n (% of column heading)	40 (6%)	40 (31%)	N/A
White (% of column heading)	481 (77%)	52 (41%)	429 (86%)
Not White (% of column heading)	28 (4%)	4 (3%)	28 (22%)
Not reported (% of column heading)	114 (18%)	72 (56%)	42 (33%)

* Minimum CA 19-9 levels (0.6 U/mL) represent the limit of detection of the COBAS instrument. N/A = not applicable.

**Table 2:** Expression levels and sensitivities of individual candidate markers when applied to the full cohort.

Marker	*p*-Value (Controls vs. Cases)	Concentration Means, Cases (CA19-9, U/mL; All Others, ng/mL)	Concentration Means, Controls (CA19-9, U/mL; All Others, ng/mL)	Sensitivity at 98% Specificity, % (95% CI)
CA 19-9	<0.001	248	12	65 (57–73)
ICAM1	<0.001	363	161	52 (44–61)
TIMP1	<0.001	657	375	48 (39–56)
CTSD	<0.001	646	333	31 (23–39)
LTBP2	<0.001	215	133	12 (6–17)
CPB1	<0.001	8139	3199	6 (2–10)
THBS1	0.220	30613	28844	2 (0–5)

## Data Availability

The original contributions presented in this study are included in the article/Supplementary Materials. Further inquiries can be directed to the corresponding author.

## Supplementary Materials

The following supporting information can be downloaded at: https://www.mdpi.com/article/10.3390/cancers17111866/s1, Table S1: Sample collection sites; Table S2: Demographics of sub-populations; Figure S1: Study participant and sample selection; Figure S2: Analyte expression as a function of age; Figure S3: Analyte expression as a function of sex; Table S3: Comparison of serum candidate protein concentrations between cases and controls in two cohorts; Table S4: Expression levels and individual sensitivities of candidate markers in cohort sub-populations; Figure S4: Density plots showing analyte distribution between cases and controls within sub-populations; Figure S5: Performance of model v2 and model v3.

## Abbreviations

The following abbreviations are used in this manuscript:

CA 19-9	Carbohydrate Antigen 19-9
CI	Confidence Interval
CPB1	Carboxypeptidase B1
CTSD	Cathepsin D
GPNMB	Glycoprotein Non-Metastatic Melanoma Protein B
ICAM1	Intracellular Adhesion Molecule 1
KLK10	Kallikrein Related Peptidase 10
LTBP2	Latent Transforming Growth Factor-Beta Binding Protein 2
MERTK	Mer Tyrosine Kinase
MMP-9	Matrix Metalloproteinase 9
NET	Neutrophil Extracellular Trap
PDAC	Pancreatic Ductal Adenocarcinoma
PLA2G1B	Phospholipase A2 group 1B
TGF-β1	Transforming Growth Factor-Beta 1
THBS1	Thrombospondin 1
TIMP1	Tissue Inhibitor of Metalloproteinase 1
