# Supplementary material for: A High Performing Biomarker Signature for Detecting Early-Stage Pancreatic Ductal Adenocarcinoma in High-Risk Individuals
Source: Cancers (Basel). 2025 Jun 2;17(11):1866. doi: 10.3390/cancers17111866 (PMC12153528; doi:10.3390/cancers17111866)
Supplement: Supplementary file 1 [file cancers-17-01866-s001.zip › Supplemental Figure S1.pdf]

A

Inclusion Criteria	Exclusion Criteria
• ≥ 40 years of age<br>• Recent diagnosis of Stage I or Stage II PDAC<br>or<br>• Participant in high-risk surveillance programs for PDAC in the United States<br>or<br>• Healthy control of any ethnicity from the United States	• Any prior treatment for PDAC including but not limited to prior resection, radiotherapy, or chemotherapy<br>• Current immunosuppressive therapy<br>• Major surgery or significant trauma within 12 weeks prior to blood sample collection<br>• Reported chronic pancreatitis<br>• Co-occurring malignancies diagnosed ≤ 3 years prior to sample collection<br>• Controls reported as symptomatic for disorders, other than diabetes, that may elevate CA 19-9 unrelated to PDAC (e.g., chronic pancreatitis, malignancies, obstructive GI abnormalities, high-grade dysplasia, mucinous cystic neoplasm of the pancreas, etc.)<br>• Sample volume insufficient for analysis (< 200 μl)

B

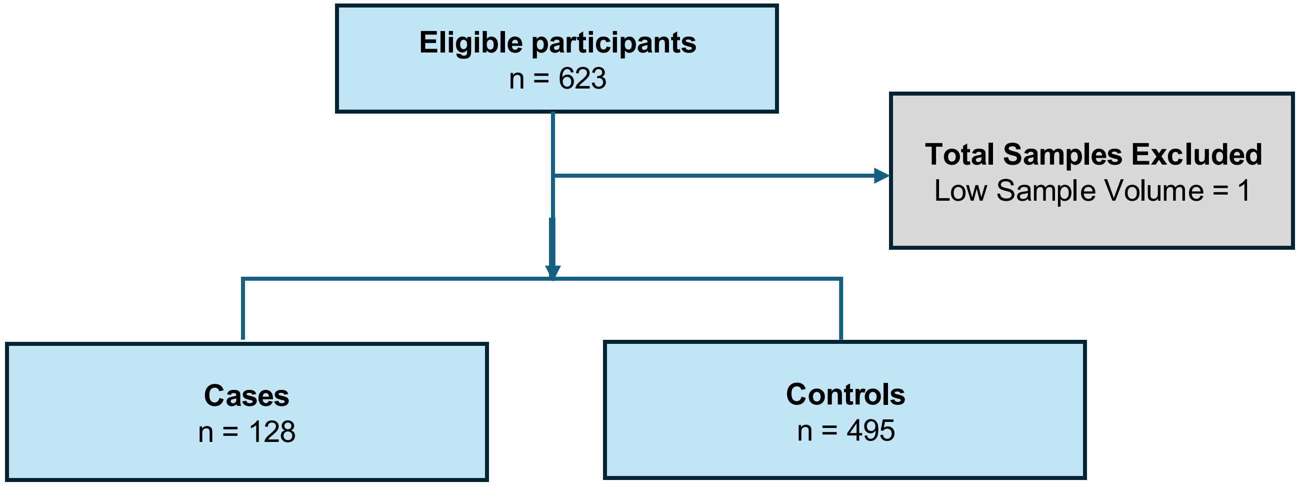

Supplemental Figure S1. Study participant and sample selection. (**A**) Inclusion and exclusion criteria for study participation. (**B**) Method of sample selection.
