# Supplementary material for: A High Performing Biomarker Signature for Detecting Early-Stage Pancreatic Ductal Adenocarcinoma in High-Risk Individuals
Source: Cancers (Basel). 2025 Jun 2;17(11):1866. doi: 10.3390/cancers17111866 (PMC12153528; doi:10.3390/cancers17111866)
Supplement: Supplementary file 1 [file cancers-17-01866-s001.zip › Supplemental Figure S2.pdf]

A

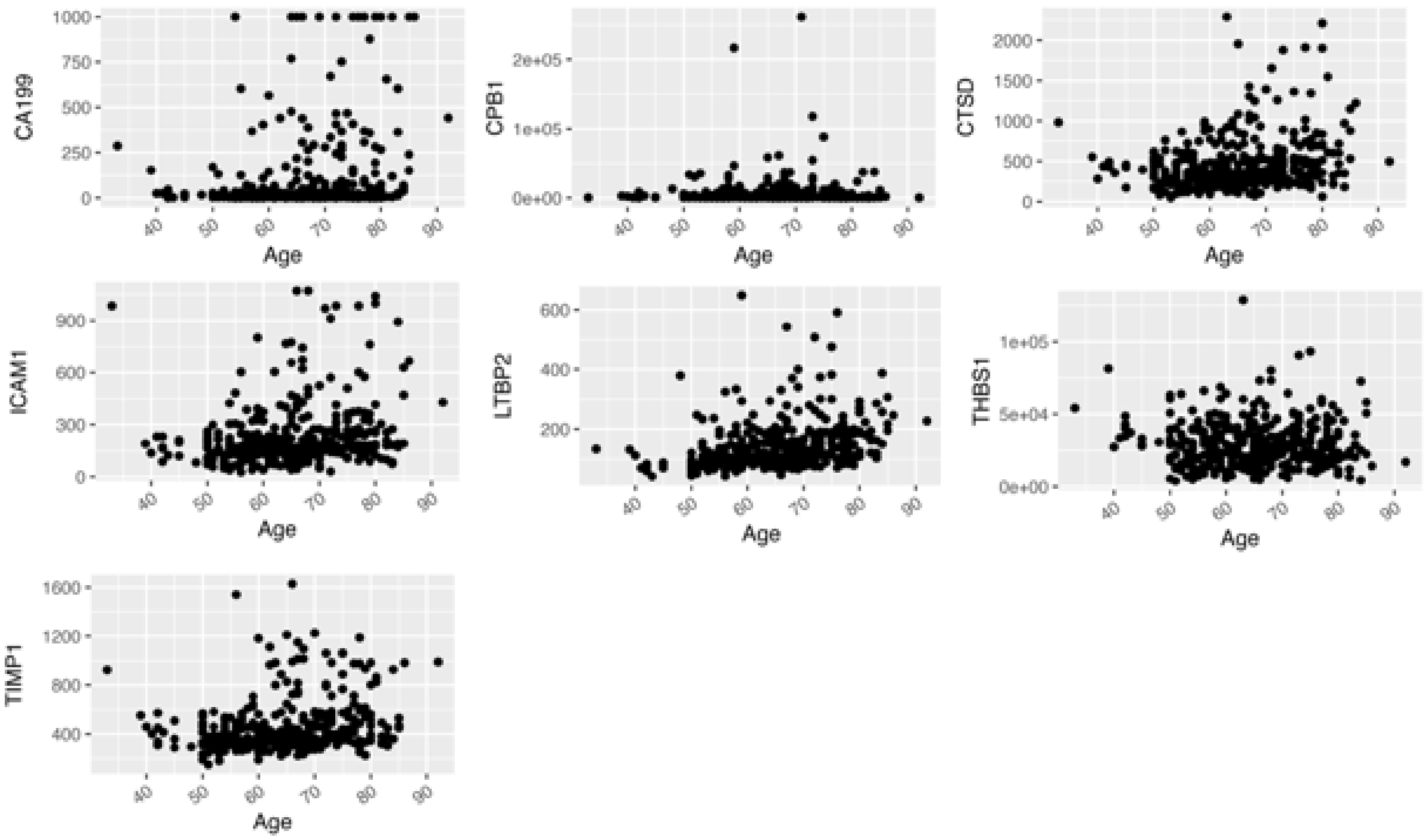

B

**Protein**	**R^2**
CA 19-9	0.043
CPB1	0.002
CTSD	0.032
ICAM1	0.037
KLK10	0.0027
LTBP2	0.104
THBS1	<0.001
TIMP1	0.042
N=623	

Supplemental Figure S2. **Analyte expression as a function of age. (A)** Scatterplots showing the amount of each candidate biomarker on the Y-axis (U/mL for CA 19-9, ng/mL for all others) and age on the X-axis. Each dot represents a single patient. **(B)** Table showing R^2 values for each analyte.
