# Supplementary material for: A High Performing Biomarker Signature for Detecting Early-Stage Pancreatic Ductal Adenocarcinoma in High-Risk Individuals
Source: Cancers (Basel). 2025 Jun 2;17(11):1866. doi: 10.3390/cancers17111866 (PMC12153528; doi:10.3390/cancers17111866)
Supplement: Supplementary file 1 [file cancers-17-01866-s001.zip › Supplemental Figure S3.pdf]

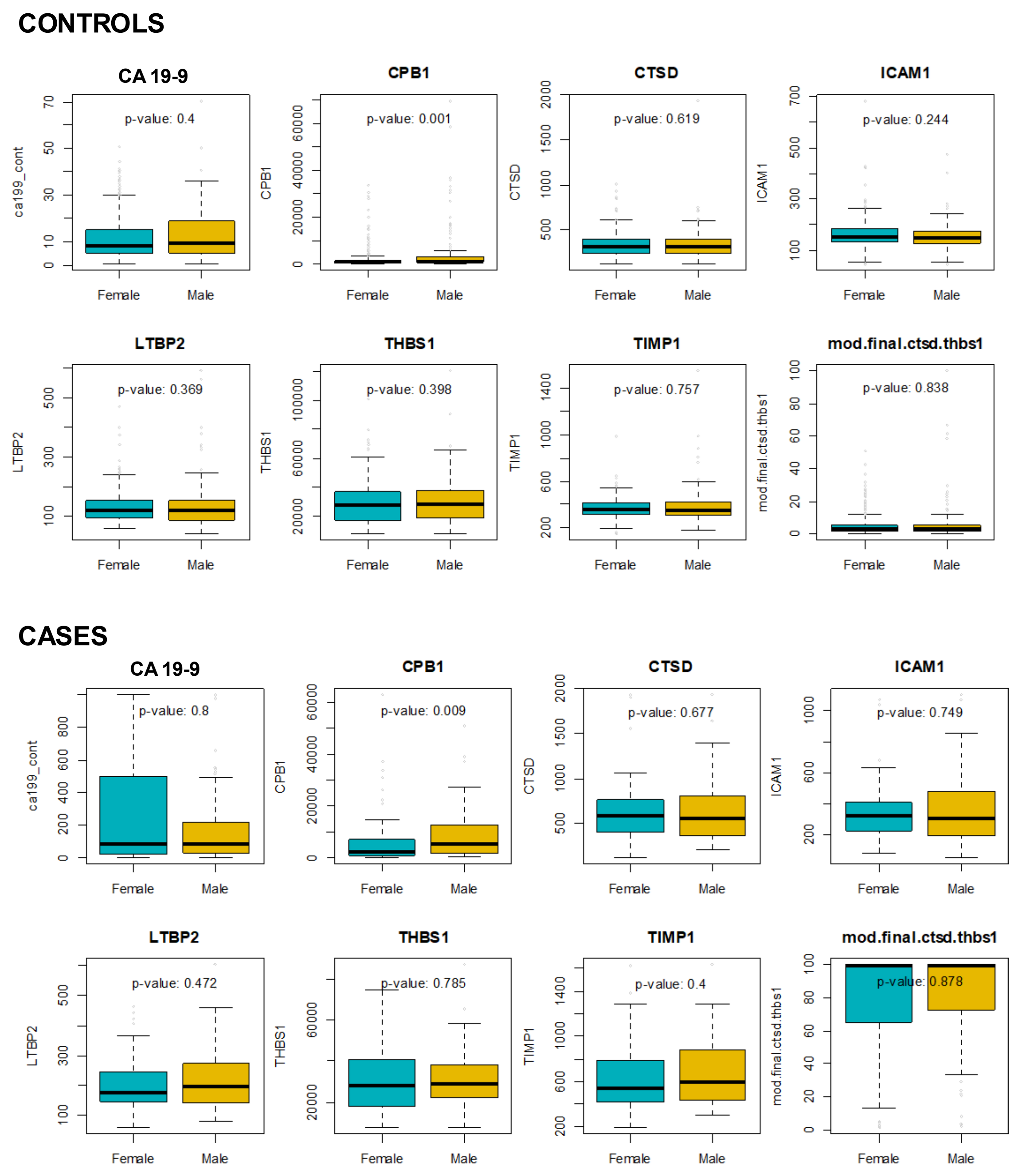

Supplemental Figure S3. **Analyte expression as a function of sex.** Boxplots comparing analyte expression between males and females in controls (**top**) and cases (**bottom**). Y-axis shows analyte amounts (U/mL for CA 19-9 and ng/mL for all others).
