# Supplementary material for: A High Performing Biomarker Signature for Detecting Early-Stage Pancreatic Ductal Adenocarcinoma in High-Risk Individuals
Source: Cancers (Basel). 2025 Jun 2;17(11):1866. doi: 10.3390/cancers17111866 (PMC12153528; doi:10.3390/cancers17111866)
Supplement: Supplementary file 1 [file cancers-17-01866-s001.zip › Supplemental Figure S4.pdf]

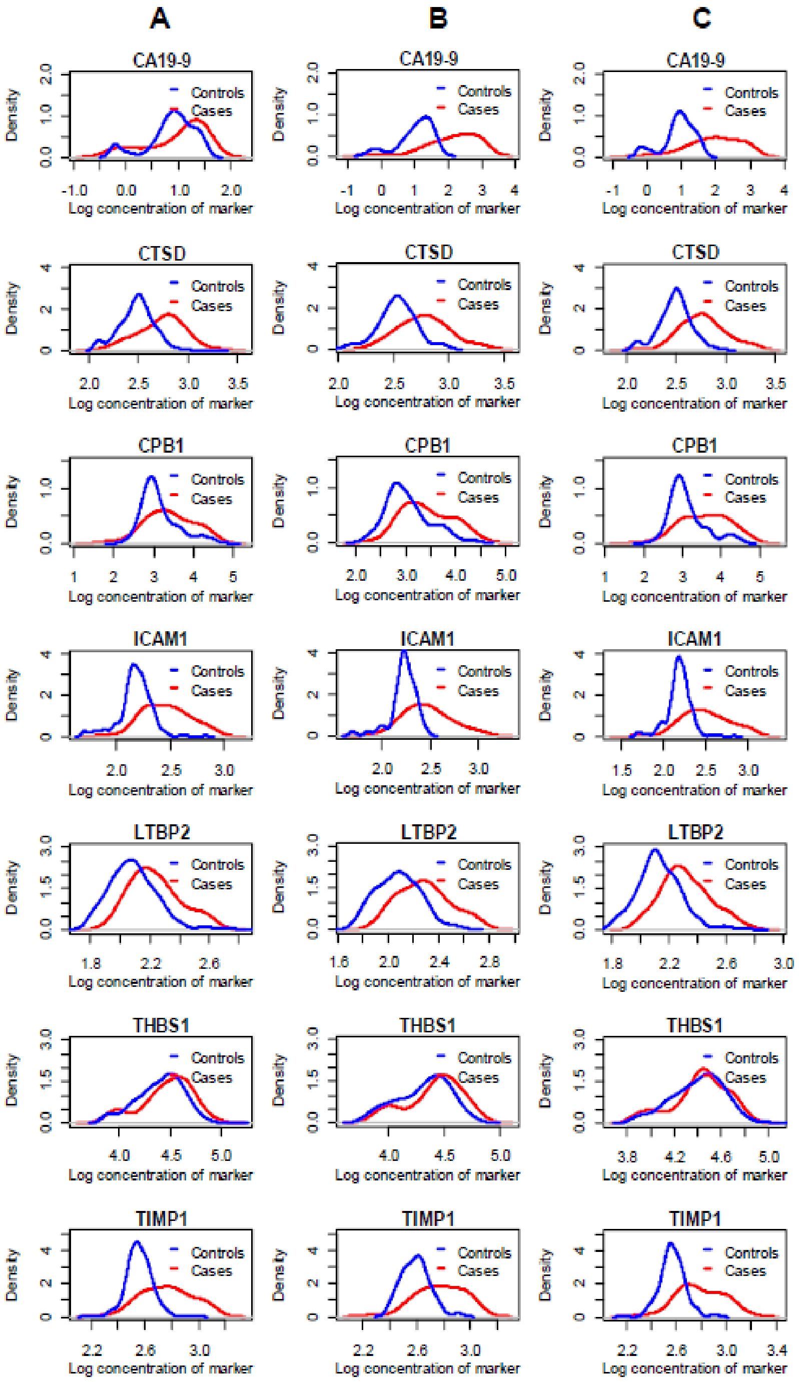

Supplemental Figure S4. Density plots showing analyte distribution between cases and controls within sub-populations. (**A**) low CA 19-9 secretors (**B**), diabetic, and (**C**) $\geq$ 65 years.
