# Supplementary material for: A High Performing Biomarker Signature for Detecting Early-Stage Pancreatic Ductal Adenocarcinoma in High-Risk Individuals
Source: Cancers (Basel). 2025 Jun 2;17(11):1866. doi: 10.3390/cancers17111866 (PMC12153528; doi:10.3390/cancers17111866)
Supplement: Supplementary file 1 [file cancers-17-01866-s001.zip › Supplemental Table S1.pdf]

**Supplemental Table S1. Sample collection sites.**

| Collection Site | Cases | Controls |
|---|---|---|
| Beth Israel Deaconess Medical Center (Boston, MA) | 13 | 0 |
| University of Chicago Medical Center (Chicago, IL) | 0 | 14 |
| Columbia University Medical Center (New York, New York) | 0 | 43 |
| Massachusetts General Hospital (Boston, MA) | 0 | 81 |
| Mount Sinai Hospital (New York, NY) | 23 | 74 |
| New York University (New York, NY) | 0 | 30 |
| Ohio State University (Columbus, OH) | 0 | 11 |
| Oregon Health & Science University Hospital (Portland, OR) | 0 | 11 |
| University of Pittsburgh Medical Center (Pittsburg, PA) | 92 | 125 |
| Stanford University Medical Center (Stanford, CA) | 0 | 16 |
| University of Massachusetts Medical School (Worcester, MA) | 0 | 11 |
| University of Pennsylvania (Philadelphia, PA) | 0 | 49 |
| | | |
| Discovery Life Sciences, Inc. (Huntsville, AL)* | 0 | 30* |

*30 normal-risk control samples were purchased from the commercial vendor Discovery Life Sciences, Inc.
