# Supplementary material for: A High Performing Biomarker Signature for Detecting Early-Stage Pancreatic Ductal Adenocarcinoma in High-Risk Individuals
Source: Cancers (Basel). 2025 Jun 2;17(11):1866. doi: 10.3390/cancers17111866 (PMC12153528; doi:10.3390/cancers17111866)
Supplement: Supplementary file 1 [file cancers-17-01866-s001.zip › Supplemental Table S2.pdf]

**Supplemental Table S2. Demographics of sub-populations.**			
**Sub-population**	All (n=623)	Cases (n=128)	Controls (n=495)
**Low CA 19-9 (< 37 U/mL)**	525 (84%)	45 (35%)	480 (97%)
**Diabetes**	91 (15%)	43 (34%)	48 (10%)
**≥ 65 years**	303 (49%)	89 (70%)	214 (43%)
