# Supplementary material for: A High Performing Biomarker Signature for Detecting Early-Stage Pancreatic Ductal Adenocarcinoma in High-Risk Individuals
Source: Cancers (Basel). 2025 Jun 2;17(11):1866. doi: 10.3390/cancers17111866 (PMC12153528; doi:10.3390/cancers17111866)
Supplement: Supplementary file 1 [file cancers-17-01866-s001.zip › Supplemental Table S3.pdf]

Supplemental Table S3. **Comparison of serum candidate protein concentrations between cases and controls in two cohorts**.

	Discovery study cohort<br>Controls: n=83, PDAC: n=75		Present study cohort<br>Controls: n=495, PDAC: n=128	
**Marker**	p-value*	up/down regulated	p-value*	up/down regulated
**CA 19-9**	<0.001	up regulated	<0.001	up regulated
**CPB1**	0.008	up regulated	<0.001	up regulated
**CTSD**	<0.001	up regulated	<0.001	up regulated
**ICAM1**	<0.001	up regulated	<0.001	up regulated
**LTBP2**	<0.001	up regulated	<0.001	up regulated
**TIMP1**	<0.001	up regulated	<0.001	up regulated
**THBS1**	0.127	not significant	0.220	not significant

*P-values compare analyte concentrations between cases and controls within the same cohort.
