# Supplementary material for: A High Performing Biomarker Signature for Detecting Early-Stage Pancreatic Ductal Adenocarcinoma in High-Risk Individuals
Source: Cancers (Basel). 2025 Jun 2;17(11):1866. doi: 10.3390/cancers17111866 (PMC12153528; doi:10.3390/cancers17111866)
Supplement: Supplementary file 1 [file cancers-17-01866-s001.zip › Supplemental Table S4.pdf]

Supplemental Table S4. Expression levels and individual sensitivities of candidate markers in cohort sub-populations.

	P-value (Controls vs. PDAC)			Sensitivity at 98% specificity, % (95%, CI)		
Marker	Low CA 19-9 (< 37 U/mL)	Diabetes	≥ 65 years	Low CA 19-9 (< 37 U/mL)	Diabetes	≥ 65 years
CA 19-9	0.276	< 0.001	< 0.001	9 (1-17)	79 (67-91)	71 (61-80)
ICAM1	< 0.001	< 0.001	< 0.001	49 (34-63)	53 (39-68)	52 (41-62)
TIMP1	< 0.001	< 0.001	< 0.001	49 (34-63)	53 (39-68)	49 (39-60)
CTSD	< 0.001	< 0.001	< 0.001	29 (16-42)	28 (15-41)	31 (22-41)
LTBP2	< 0.001	< 0.001	< 0.001	9 (1-17)	26 (13-39)	9 (3-15)
CPB1	0.040	0.006	<0.001	2 (0-7)	23 (11-36)	10 (4-16)
THBS1	0.068	0.031	0.414	4 (0-10)	9 (1-18)	1 (0-3)

Expression levels that were not significantly different are shown in gray.
