# Supplementary material for: A High Performing Biomarker Signature for Detecting Early-Stage Pancreatic Ductal Adenocarcinoma in High-Risk Individuals
Source: Cancers (Basel). 2025 Jun 2;17(11):1866. doi: 10.3390/cancers17111866 (PMC12153528; doi:10.3390/cancers17111866)
Supplement: Supplementary file 1 [file cancers-17-01866-s001.zip › Supplemental Figure S5.pdf]

A.

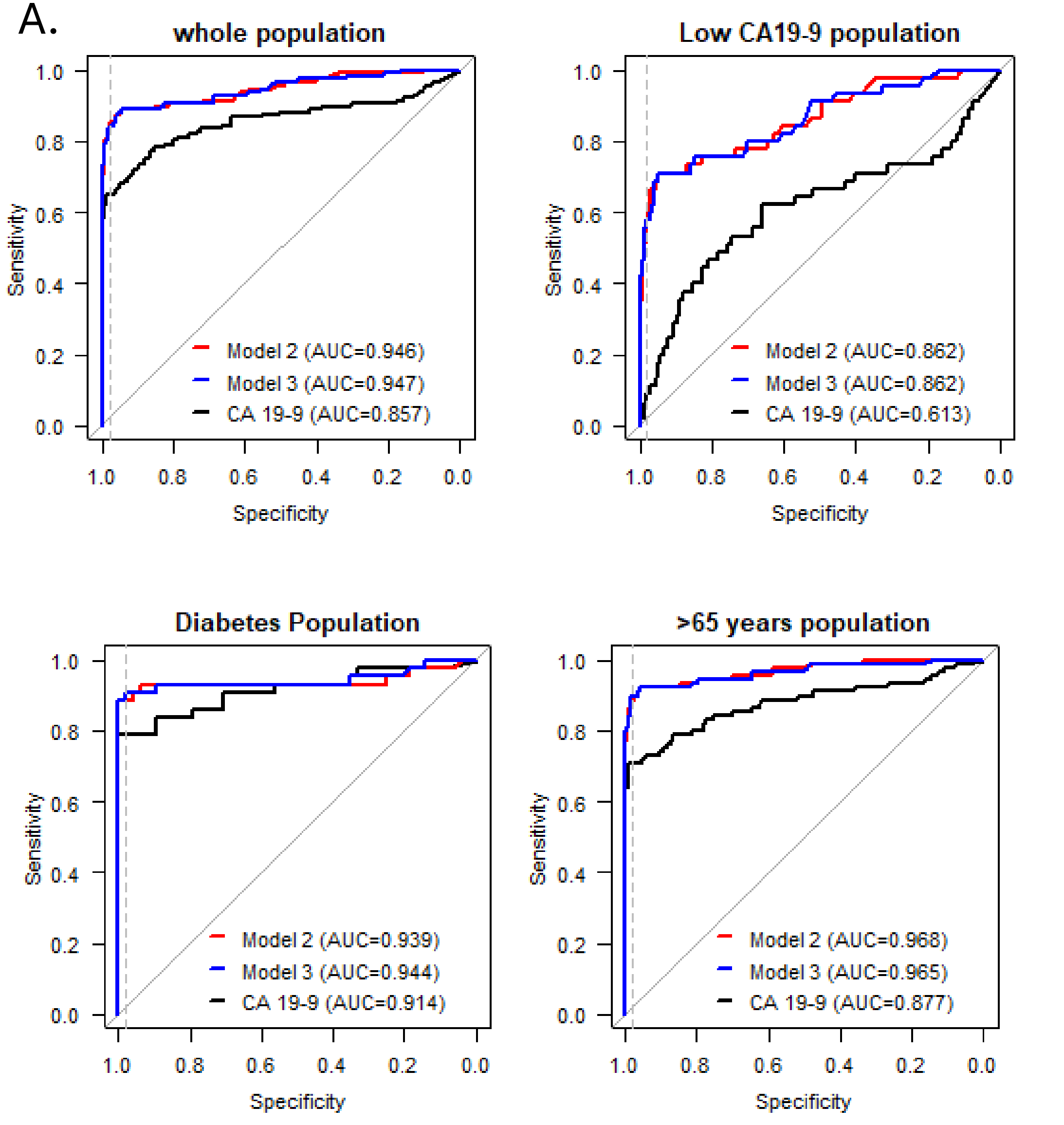

B.

	Full Cohort	Low CA 19-9	Diabetes	≥ 65 years
**Model**	Sensitivity, % (95% CI)			
**Model v2: CA 19-9 + TIMP1 + ICAM1 + CTSD**	84 (78-91)*	58 (43-72)*	91 (82-99)*	84 (77-90)*
**Model v3: CA 19-9 + TIMP1 + ICAM1 + THBS1**	84 (77-90)*	56 (41-70)*	91 (82-99)*	90 (84-96)*
**CA 19-9**	65 (57-73)	9 (1-17)	79 (67-91)	71 (61-80)

C.

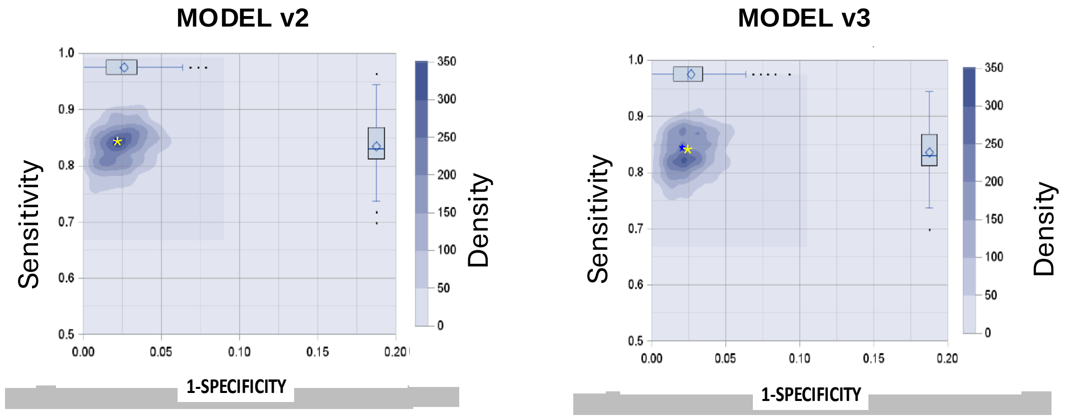

Supplemental Figure s5 Performance of model v2 and model v3. (A) ROC curves of both versions of the model compared to CA 19-9 alone in the full cohort and sub-populations. Gray lines (comparators) depict no model discrimination between groups (AUC = 0.5). **(B)** Sensitivity of each model and CA 19-9 at 98% specificity presented as a percentage with a 95% CI. Both versions of the model demonstrate significantly higher sensitivities in the full cohort and all sub-populations compared to CA 19-9 alone. *p < 0.001. **(C)** Density plots and boxplots of the 1000x random splits showing robustness of model v2 and model v3. The yellow stars indicate model performance when trained using the full cohort.
